# Validity and reliability of a food frequency questionnaire to estimate dietary intake among Lebanese children

**DOI:** 10.1186/s12937-015-0121-1

**Published:** 2016-01-12

**Authors:** Patricia Moghames, Nour Hammami, Nahla Hwalla, Nadine Yazbeck, Hikma Shoaib, Lara Nasreddine, Farah Naja

**Affiliations:** 1 Department of Nutrition and Food Sciences, Faculty of Agricultural and Food Sciences, American University of Beirut, Lebanon, 11-0236 Riad El Solh, 1107-2020 Beirut, Lebanon; 2 School of Public Health and Health Systems, Faculty of Applied Health Sciences, University of Waterloo, 200 University Avenue West, Waterloo, ON N2L 3G1 Canada; 3 Department of Pediatrics and Adolescent Medicine, American University of Beirut Medical Center, Lebanon, 11-0236 Riad El Solh, 1107-2020 Beirut, Lebanon

**Keywords:** Food frequency questionnaire, School-age children, Validation, Calibration, Lebanon

## Abstract

**Background:**

Nutritional status during childhood is critical given its effect on growth and development as well as its association with disease risk later in life. The Middle East and North Africa (MENA) region is experiencing alarming rates of childhood malnutrition, both over- and under-nutrition. Hence, there is a need for valid tools to assess dietary intake for children in this region. To date, there are no validated dietary assessment tools for children in any country of the MENA region. The main objective of this study was to examine the validity and reliability of a Food Frequency Questionnaire (FFQ) for the assessment of dietary intake among Lebanese children.

**Methods:**

Children, aged 5 to 10 years (*n* = 111), were recruited from public and private schools of Beirut, Lebanon. Mothers (proxies to report their children’s dietary intake) completed two FFQs, four weeks apart. Four 24-hour recalls (24-HRs) were collected weekly during the duration of the study. Spearman correlations and Bland-Altman plots were used to assess validity. Linear regression models were used to derive calibration factors for boys and girls. Reproducibility statistics included Intraclass Correlation Coefficient (ICC) and percent agreement.

**Results:**

Correlation coefficients between dietary intake estimates derived from FFQ and 24-HRs were significant at *p* < 0.001 with the highest correlation observed for energy (0.54) and the lowest for monounsaturated fatty acids (0.26). The majority of data points in the Bland-Altman plots lied between the limits of agreement, closer to the middle horizontal line. After applying the calibration factors for boys and girls, the mean energy and nutrient intakes estimated by the FFQ were similar to those obtained by the mean 24-HRs. As for reproducibility, ICC ranged between 0.31 for *trans*-fatty acids and 0.73 for calcium intakes. Over 80 % of study participants were classified in the same or adjacent quartile of energy and nutrients intake.

**Conclusions:**

Findings of this study showed that the developed FFQ is reliable and is also valid, when used with calibration factors. This FFQ is a useful tool in dietary assessment and evaluation of diet-disease relationship in this age group.

**Electronic supplementary material:**

The online version of this article (doi:10.1186/s12937-015-0121-1) contains supplementary material, which is available to authorized users.

## Background

Nutritional status during childhood has been shown to have profound effects on overall health and chronic disease development [1, 2]. On one hand, caloric deficit and nutritional deficiencies have detrimental effects on growth, mental development, and quality of life [3, 4]. Under-nutrition is postulated to contribute to 35 % of child deaths per year [4]. On the other hand, over-nutrition and childhood obesity, together with genetic predisposition and other environmental and lifestyle factors have been associated with an increasing risk of adult-onset diseases such as type 2 diabetes, cardiovascular disease, hypertension and polycystic ovarian syndrome [5, 6]. Furthermore, it is during early life that children acquire dietary habits that persist through adulthood [7, 8]. Validated tools to assess dietary intake among children are essential to provide reliable data on their nutritional status and to develop effective evidence-based strategies and programs to address diet-related problems in this young age group.

Various methods have been developed in order to assess dietary intake, ranging from relatively simple techniques such as 24-h dietary recalls, estimated or weighed food records, narrative diet histories and FFQ, to the more complex biochemical approach of measuring markers of nutrient intakes in blood, urine or other biological samples. Though it is argued that weighed diet records and biomarkers are the ‘gold standards’ of dietary intake assessment, each of the various methods of dietary assessment presents a distinct set of advantages, associated errors and practical difficulties to be considered [9]. Although food records and 24-hour recalls (24-HRs) are commonly used, time, literacy, and economic constraints of these methods render them unsuitable for large-scale studies [10]. Food Frequency Questionnaires (FFQs), on the other hand, are considered to be more practical and economical for the collection of dietary data in large-scale epidemiological studies for they are easier to administer, less intrusive, less time-consuming, relatively inexpensive and less burdensome on both interviewers and participants [10, 11]. In addition, unlike 24-HRs and food records, which are both considered as short term dietary assessment tools, FFQs cover a longer period of dietary recall varying from months to years [12]. Measuring average long-term diet may be more valuable than measuring the intake of few specific days, particularly when aiming to assess the relation between food and related long-latent diseases with modifiable risk factors, such as obesity and diet related non-communicable diseases [10].

The accuracy of dietary intake information generated by an FFQ is highly dependent on the validity and reproducibility of this FFQ in the population it is intended to be used for [13]. FFQs aiming to assess dietary intake by nutrients and/or food groups have been validated in all age groups for more than 20 years in countries worldwide [11, 14, 15]. In the Middle East and North Africa (MENA) region, FFQs have been validated among adults in the Islamic Republic of Iran [16], United Arab Emirates and Kuwait [17], and Jordan [18]. However, to date, there has been no validated FFQ to assess dietary intake among children in the MENA region. Such a situation has hampered research to identify children population groups at risk of malnutrition and has attenuated the validity of studies on diet-disease associations among children in the region. According to estimates by the United Nations Children’s Fund, the MENA region is witnessing a double burden of child malnutrition concomitant with a state of nutrition transition [19], whereby a gradual shift towards a more westernized type of dietary intake is taking place, together with the erosion of the traditional diets, rich in fruits and vegetables [20]. Children represent a vulnerable group whose nutritional status and dietary intake could be most affected by this transition [21]. Therefore the main aim of this study is to validate a culturally sensitive FFQ for the assessment of dietary intake among Lebanese children. More specifically, the objectives of this study are to 1) develop an FFQ with a culturally appropriate list of foods commonly consumed among Lebanese children, 2) determine the relative validity of the developed FFQ in measuring energy and nutrient intakes as compared to the means obtained by repeated 24-HRs 3) calibrate the FFQ using linear calibration factors and 4) evaluate the reproducibility of this FFQ.

## Methods

### Study participants

Children aged between 5 and 10 years were recruited from public and private schools in the Greater Beirut area, Lebanon, following a random cluster sampling design. Fourteen schools were randomly selected from the Ministry of Education list of schools in the Greater Beirut area (*n* = 32). In the selected schools, all students aged between 5 and 10 years received an invitation letter to participate in the study, addressed to their parents. In this letter the eligibility criteria (inclusion/exclusion) to participate in the study were outlined. These criteria were: Parents holding the Lebanese nationality or residing in Lebanon for more than 10 years, and children to be healthy (i.e., with no medical conditions, allergies nor specific dietary restrictions affecting food intake). Interested parents were contacted by trained research dietitians and were enrolled in the study between October 2011 and June 2012. Data collection was conducted between October and July, hence covering the various seasons in Lebanon. The study protocol was approved by the Institutional Review Board of the Social and Behavioral Sciences at the American University of Beirut, Lebanon. Participating parents gave a written informed consent and children above the age of seven also signed an informed assent to indicate their approval to participate in the study. All parents who signed the consent form were the mothers of the children.

### Study protocol

**Fig. 1:**
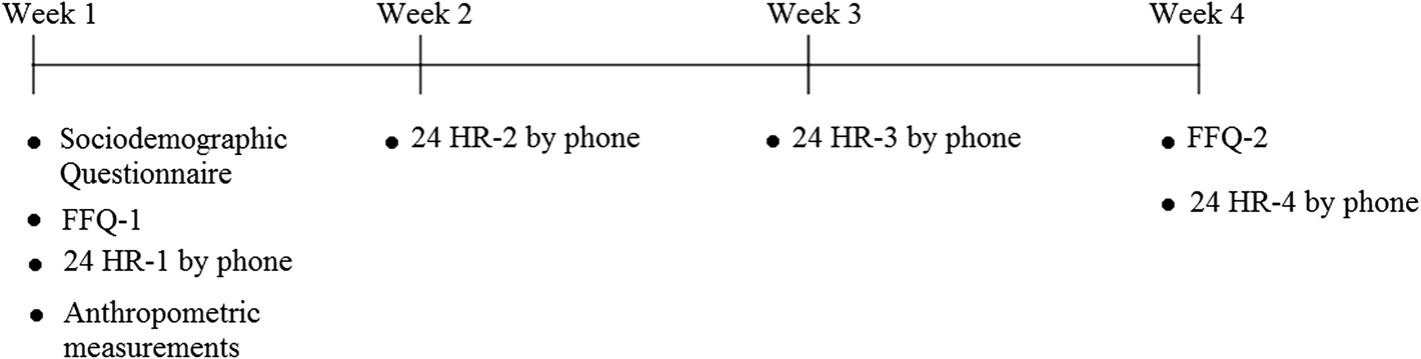
A schematic representation of the study protocol aiming to validating a Food Frequency Questionnaire (FFQ) against 24-hour recalls (24-HRs) among school-aged children in Lebanon

Participants were enrolled in the study for a period of 4 weeks (Fig. 1), during which two face-to-face interviews took place at the Nutrition and Food Sciences department at the American University of Beirut. In the first face to face interview with the child and his/her mother, a socio-demographic questionnaire and the first FFQ (FFQ-1) were completed. In addition anthropometric measurements of the child were obtained. After four weeks, another face to face interview took place with the mother and the child whereby a second FFQ (FFQ-2) was completed. Four 24-HRs of the child’s diet were collected by phone. These 24-HRs were one week apart between the two interviews. For both dietary intake collection methods (FFQ and 24-HR), information was collected from the mother, in the presence of the child. For each participant, all interviews (face to face and phone) were carried by the same research dietitian. Mothers were constantly encouraged to maintain their children’s regular dietary habits. Probing and interviewing techniques were standardized to minimize interviewer bias.

Out of the 171 mothers who returned the signed invitation letter indicating their interest to participate, 7 were excluded as they were not eligible according to the inclusion/exclusion criteria of the study. Out of the remaining 164 mother-child pair, 125 showed up for their first visit. The total number of participants who completed the study was 120 (dropout rate: 4 %). The main reasons for not completing the study were lack of time and interest. This sample size is considered appropriate for studies validating dietary intake tools [10].

### Data collection

#### Socio-demographic questionnaire

The socio-demographic questionnaire included information about the mother’s age, education, marital status, occupation, and crowding index. In addition, the child’s age, gender and school class level were recorded.

#### Anthropometric assessment

Anthropometric measurements of the child were obtained using standardized techniques and calibrated equipment. The InBody 230 (Biospace, Korea) and stadiometers (Seca model 213, Germany) were used to measure weight (in kg) and height (in cm), respectively. Before stepping on the InBody 230, children were asked to remove as much outerwear as possible, as well as their shoes and socks and to empty their pockets. Subjects were weighed to the nearest 0.1 kg. Height was measured to the nearest 0.1 cm with the child bare footed, using a stadiometer. Body Mass Index was calculated as weight (kg)/height (m^2^). All measurements were carried in duplicates and the average was used in the analysis.

#### Dietary intake assessment

A semi-quantitative FFQ was developed to assess dietary intake among school-aged children in Lebanon. The FFQ included three sections: the food list, the portion size and the frequency response. A multitude of approaches was followed in order to compile the food list for this questionnaire: 1) a review of previously collected 24-HRs data pertaining to a national representative sample of Lebanese children 6–10 years old was carried out (*n* = 200) [22]. Frequently cited food items in the 24-HRs (>5 %) were included in the food list of the developed FFQ, 2) the developed FFQ’s food list was checked by 30 mothers of 5–10 year old children for clarity, user-friendliness as well as the cultural sensitivity/appropriateness of the food items included, and 3) the food list was also compared to previously published FFQs aiming to assess dietary intake among young children. The final FFQ food list included a total of 112 food items.

For portion size, mothers were given the option to indicate their child’s food intake in function of a reference portion size or in grams. The reference portion for each food item represented one standard serving expressed in household measures (cups, spoons and plates) and/or customary packing size. In order to assist in quantifying the reference portion size, the standard two-dimensional food portion visual chart was also used. This chart has been developed by Nutrition Consulting Enterprises and validated for use amongst adult men and women aged 20 to 70+ years as part of the Framingham Heart Study, especially for the use of telephone dietary interviewing [23]. The frequency of the child’s food intake was indicated by how many times per day, week or month the child has consumed the food. Seasonal adjustments were considered for food items that were eaten at specific times of the year. The reported frequency of consumption for these seasonal foods was adjusted for the length of the specific season they were consumed within, in order to obtain frequency of their consumption over the course of a year. For all food items in the FFQ, the frequency per day was multiplied by the portion size of the food item in order to calculate the total amount of food consumed per day.

In addition to the 112 food items, the FFQ included an open-ended section in which participants may provide information on additional foods or beverages consumed on a regular basis and which do not appear on the questionnaire’s food list. When completing the FFQ, mothers were asked to refer to their child’s diet during the 12 months prior to the interview. The completion of the FFQ lasted for approximately 30 min.

The 24-HRs dietary recalls were carried using the Multiple Pass Food Recall (MPR) 5-step approach, developed by the United States Department of Agriculture (USDA) [24]. This approach has consistently showed attenuation in the 24-HRs’ limitations [24, 25]. The five steps followed included 1) quick food list recall, 2) forgotten food list probe 3) time and occasion at which foods were consumed, 4) detailed overall cycle and 5) final probe review of the foods consumed. The four 24-HRs collected represented three weekdays, in which the child attended a regular day at school, and one weekend day (either Saturday or Sunday). For each 24-HR, the research dietitian obtained information related to the time of each meal’s intake, the food consumed by the child, its portion size, preparation methods, and the brand of the food and beverages consumed, if applicable. In order to maintain regular eating habits for their children, mothers were unaware of the day the 24-HR would be conducted. Upon calling, the interviewer made sure that the child was present with the mother during the completion of the 24-HRs as the child was probed and asked details of his/her intake in case of foods he/she has eaten away from home such as in the school. The mean of dietary intakes estimated by the four 24-HRs were used as the reference method against which the FFQ was validated.

The Nutritionist Pro software (version 5.1.0, 2014, First Data Bank, Nutritionist Pro, Axxya Systems, San Bruno, CA) was used for the analysis of the dietary intake data and to estimate energy, macro- and micronutrients’ intakes. For composite and mixed dishes, standardized recipes were added to the Nutritionist Pro Software using single food items. Within the Nutritionist Pro, the USDA database was selected for analysis (SR 24, published September 2011). Food composition of specific Lebanese foods (not included in the Nutritionist Pro software database) was obtained from local food composition tables [26].

### Statistical analysis

Frequencies and percentages as well as means and standard deviations (SD) were used to describe categorical and continuous variables, respectively. For the validity assessment of the FFQ, dietary intakes derived from the FFQ-1 were compared to the mean of the four 24-HRs using energy-adjusted Spearman correlation coefficient (*r*). Energy adjustment was carried out using the residual method as per Willet et al. [27]. Distribution of the study participants according to quartiles of intake was calculated and the degree of agreement between FFQ-1 and mean 24-HRs was evaluated using contingency tables of quartiles. Furthermore, the analysis proposed by Bland & Altman [28] was used to graphically examine the agreement between the two methods. For this analysis, the difference in intake between the two methods (FFQ-1 and mean 24-HRs) was plotted against the mean intake of the two measures ((FFQ-1+ mean 24-HRs)/2). The plots include lines for the mean difference and the Limits of Agreement (LOA), defined as mean difference ± 1.96 x SD. The calibration of the FFQ was conducted as per the method described by Araujo et al. [29] and which involved the derivation of calibration coefficients relating dietary intakes estimated from the reference method (mean 24-HRs) to the test method (FFQ-1). These coefficients are obtained from linear regression equations using dietary intake values from mean 24-HRs as dependent variables and those of the FFQ-1 as independent variables. The regression constant (α) and the slope of regression (β) were estimated. The calibrated values for each nutrient were estimated based on α and β coefficients using the following formula:

$$ \mathrm{Calibrated}\ \mathrm{dietary}\ \mathrm{intakes} = \upalpha +\upbeta \kern0.5em \mathrm{F}\mathrm{F}\mathrm{Q}\hbox{-} 1 $$

where, α: regression constant; β: slope of regression; FFQ-1: Dietary intakes as estimated by FFQ-1.

For the reproducibility of the FFQ, Spearman correlation (adjusted for energy) between FFQ-1 and FFQ-2 was used. In addition, the Intraclass Correlation Coefficient (ICC) was calculated to examine the agreement between FFQ-1 and FFQ-2 in ranking individuals according to their energy, macro- and micronutrients intake. The degree of agreement between FFQ-1 and FFQ-2 was further evaluated using contingency tables of quartiles and weighted Kappa (κw) test with values of κw between 0.40 and 0.59 considered moderate, 0.60 to 0.79 substantial and 0.8 outstanding as per Landis & Koch [30].

Data entry was carried out using Statistical Package for Social Sciences 22.0 (SPSS for Windows, 2013, Chicago: SPSS Inc.). A *p*-value less than 0.05 was considered statistically significant. In this manuscript, the results for energy and selected nutrients are presented (energy, proteins, carbohydrates, fats, monounsaturated fatty acids (MUFA), polyunsaturated fatty acids (PUFA), *trans*-fatty acids, calcium, iron, fiber, and sugar). Results pertinent to the remaining micronutrients are found in Additional file 1.

## Results

After examination of outliers (energy intake <500 and >4700 Kcal/day), 9 subjects were excluded (7 males and 2 females), leaving a total of 111 participants to be included in the analysis of this study. The energy intake cut offs used for detection of outliers were calculated using mean ± 2.5 SDs, using data derived from the mean 24-HRs. Table 1 displays the socio demographic characteristics and anthropometric measurements of the study participants, both mothers and children. Fifty-eight girls and 53 boys took part in this study, with an average age of 8.15 years (SD 2.04) and with the majority of participants coming from private schools (92.8 %). Using the WHO cut-offs of BMI z-scores [31], over half of the children (52.2 %) surveyed were either overweight (18.9 %) or obese (33.3 %). The mean age for the mothers was 38.45 years (SD 6.36), with only 26 % having an education less than high school, while 46 % had a university degree. Most of the mothers were married (93.3 %) and only 28.8 % of mothers were employed.

**Table 1:** Socio-demographic characteristics and anthropometric measurements of study participants (*n* = 111)^a^

Socio-demographic variables	Total	Females (*n* = 58)	Males (*n* = 53)
Age of mother (years)	38.45 ± 6.36	38.95 ± 6.87	37.91 ± 5.76
Age of child (years)	8.15 ± 2.04	8.09 ± 2.02	8.23 ± 2.07
Gender		58 (52.3)	53 (47.7)
School			
Private	63 (56.8)	33 (56.9)	30 (56.6)
Public	48 (43.2)	25 (43.1)	23 (43.4)
Grade level			
Kindergarten	6 (5.4)	4 (6.9)	2 (3.77)
Grade 1	30 (27.0)	13 (22.4)	17 (32.1)
Grade 2	17 (15.3)	10 (17.2)	7 (13.2)
Grade 3	17 (15.3)	10 (17.2)	7 (13.2)
Grade 4	24 (21.6)	13 (22.4)	11 (20.8)
Grade 5	6 (5.4)	2 (3.4)	4 (7.5)
Grade 6	11 (10.0)	6 (10.3)	5 (9.4)
Mother’s marital status			
Divorced, separated or widowed	8 (6.7)	4 (6.9)	4 (7.5)
Married	112 (93.3)	54 (93.1)	49 (92.5)
Mother’s educational level			
Less than high school degree	26 (23.4)	16 (27.6)	10 (18.9)
Attained a high school degree	39 (35.2)	16 (27.6)	23 (43.4)
Attained a university degree	46 (41.4)	26 (44.8)	20 (37.7)
Mother’s employment status			
Not employed	79 (71.2)	40 (69.0)	39 (73.6)
Employed	32 (28.8)	18 (31.0)	14 (26.4)
Crowding index	1.27 ± 0.66	1.34 ± 0.79	1.20 ± 0.47
<1	56 (50.5)	30 (51.7)	26 (49.1)
1.00–1.50	29 (26.1)	14 (24.1)	15 (28.3)
>1.50	26 (23.4)	14 (24.1)	12 (22.6)
Anthropometric measurements of the child			
Body Mass Index (BMI) (kg/m2)^b^			
Normal weight (−2–0.99 SD)	53 (47.7)	29 (50.0)	24 (45.3)
Overweight (1–1.99 SD)	21 (18.9)	13 (22.4)	8 (15.1)
Obese (≥2.00 SD)	37 (33.3)	16 (27.6)	21 (39.6)

^a^Numbers in this table represent means ± standard deviations (SD) and n (%) for continuous and categorical variables, respectively

^b^BMI cut-off points for children adapted from Wang & Chen [33]

**Fig. 2:**
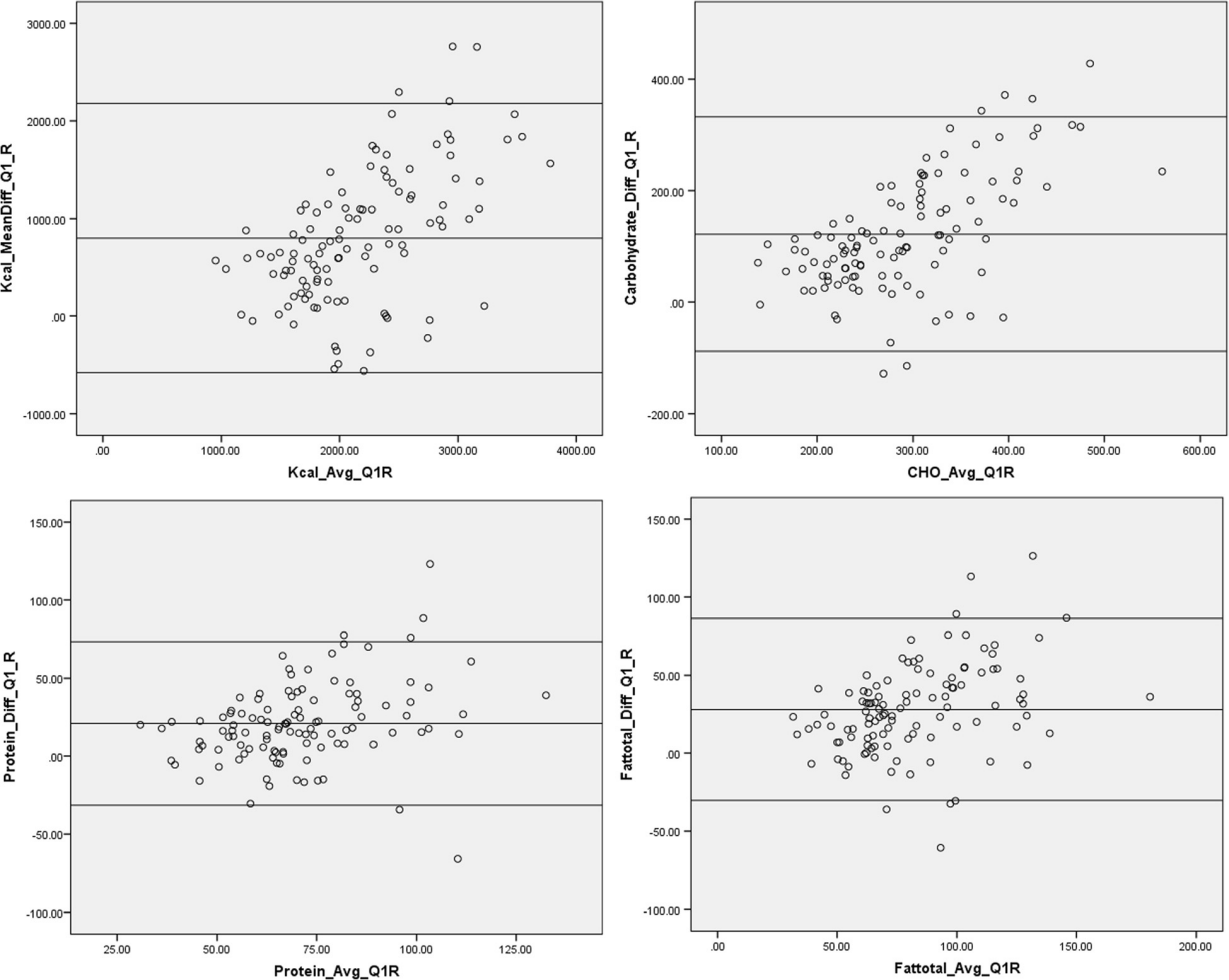
Bland-Altman plots for energy and selected nutrients (carbohydrates, protein, total fat) as predicted by the first Food Frequency Questionnaire (FFQ-1) and the mean of four 24-hour recalls (24-HRs)

The mean intake of energy and selected nutrients as measured by FFQ-1 and 24-HRs are shown in Table 2. The mean difference, Spearman’s *r* (adjusted for energy), percent agreement and LOAs between the two methods are also presented in this table. Energy and nutrients’ intake obtained by the FFQ were higher than those reported by the mean 24-HRs for energy and all nutrients considered in this study. Spearman’s *r* between the two methods were statistically significant at *p* < 0.001 with the highest correlation observed for energy (0.54) and the lowest for MUFA (0.26). Over 75 % of subjects were classified in the same and/or adjacent quartile of energy and nutrients intakes derived from FFQ-1 and 24-HRs. The Bland-Altman plots for energy, protein, carbohydrates, and fat are presented in Fig. 2. With the exception of a few, the majority of the data points lied between the LOAs, closer to the middle horizontal line (Table 2).

**Table 2:** Mean ± SD, mean difference, Spearman’s correlation (*r*), % agreement and 95 % Limits of Agreement (LOA) for selected energy and nutrients intakes as measured by FFQ-1 and mean 24-HRs (*n* = 111)

Nutrients	FFQ-1	24-HRs	Mean difference	Spearman’s r^a^	Percent agreement (same & adjacent quartile)	95 % LOA	
	Mean ± SD	Mean ± SD					
Energy (Kcal)	2560.86 ± 822.32	1760.66 ± 489.26	800.20 ± 689.88	0.54**	81.08	−1015.12	−170.37
Protein (g)	81.71 ± 25.88	60.71 ± 19.23	21.00 ± 26.22	0.33**	78.38	−28.36	9.03
Carbohydrate (g)	351.87 ± 120.07	229.87 ± 63.52	121.99 ± 105.15	0.38**	81.98	−170.79	−52.54
Fat (g)	96.47 ± 35.70	68.42 ± 25.82	28.04 ± 29.15	0.33**	84.68	−13.35	−2.16
Saturated fat (g)	30.17 ± 12.47	21.00 ± 7.49	9.17 ± 11.06	0.39**	81.98	−14.16	7.89
MUFA (g)	34.87 ± 13.31	24.20 ± 10.02	10.67 ± 10.92	0.26**	87.39	−5.58	6.13
PUFA (g)	20.80 ± 10.35	15.89 ± 9.92	4.91 ± 9.99	0.33**	81.08	−0.48	8.25
*Trans*-fatty Acid (g)	0.16 ± 0.16	0.10 ± 0.10	0.05 ± 0.16	0.34**	76.58	−0.06	0.02
Calcium (mg)	996.76 ± 365.68	628.19 ± 231.70	368.56 ± 364.47	0.44**	79.28	−382.53	44.68
Iron (mg)	19.09 ± 7.15	13.37 ± 4.65	5.72 ± 5.83	0.45**	80.18	−6.00	0.28
Fiber (g)	23.36 ± 9.60	15.00 ± 5.03	8.36 ± 8.77	0.37**	79.28	−12.12	−3.61
Sugar (g)	113.47 ± 47.94	63.28 ± 21.72	50.24 ± 43.25	0.35**	84.68	−55.86	−19.57

**P value < 0.01

^a^Adjustment for energy was carried using the residual method

For the calibration, the regression equations initially included age and sex as covariates. However, since the regression constants for sex were significant, the calibration analyses were carried separately for boys and girls. Age was not found to significantly affect the association between the dependent and independent variables in the regression analysis and hence it was deleted. The calibration coefficients (α and β) and the calibrated mean intakes of energy and selected nutrients are presented in Table 3. For girls, β ranged between 0.23 for *trans*-fatty acids to 0.72 for PUFA. As for boys, the lowest value for β was observed for sugar (0.09) and the highest for iron intakes (0.43). Using these coefficients, the mean calibrated values of FFQ-1 were found to be similar to the test method (24-HRs) for energy and all of the selected nutrients (Table 3).

**Table 3:** Calibration parameters and means ± (SD) for energy and nutrients intake estimated from FFQ-1, 24-HRs and calibrated FFQ-1 for boys and girls (*n* = 111)

Energy/nutrients	α (95 % CI)	β (95 % CI)	FFQ-1	24-HRs	Calibrated FFQ-1
			Mean ± SD	Mean ± SD	Mean ± SD
Girls (*n* = 58)					
Energy (Kcal)	644.56 (332.25–956.88)	0.43 (0.31–0.55)**	2455.59 ± 809.79	1695.71 ± 503.88	1695.55 ± 346.59
Protein (g)	36.38 (20.12–52.65)	0.28 (0.08–0.49)**	77.49 ± 23.74	58.49 ± 19.06	58.47 ± 6.76
Carbohydrate (g)	107.21 (67.96–146.46)	0.33 (0.22–0.44)**	334.74 ± 114.28	217.65 ± 60.58	217.67 ± 37.71
Fat (g)	17.70 (1.75–33.64)	0.53 (0.37–0.68)**	94.40 ± 37.46	67.54 ± 29.58	67.73 ± 19.85
Saturated fat (g)	8.03 (3.31–12.74)	0.42 (0.26–0.58)**	28.06 ± 10.77	19.80 ± 7.77	19.78 ± 4.51
MUFA (g)	7.14 (1.06–13.22)	0.48 (0.32–0.65)**	34.74 ± 14.75	23.97 ± 11.40	23.81 ± 7.08
PUFA (g)	1.31 (–3.78–6.40)	0.72 (0.50–0.93)**	21.04 ± 10.78	16.43 ± 11.64	16.43 ± 7.75
*Trans*-fatty acid (g)	0.07 (0.03–0.10)	0.23 (0.08–0.38)*	0.15 ± 0.17	0.10 ± 0.10	0.10 ± 0.04
Calcium (mg)	342.33 (181.53–503.14)	0.24 (0.08–0.41)*	921.57 ± 278.95	566.81 ± 186.94	567.19 ± 68.06
Iron (mg)	6.93 (3.91–9.95)	0.32 (0.17–0.48)**	18.67 ± 6.88	13.00 ± 4.50	13.00 ± 2.23
Fiber (g)	8.77 (5.78–11.77)	0.24 (0.12–0.36)**	23.07 ± 9.80	14.30 ± 4.97	14.31 ± 2.35
Sugar (g)	30.10 (18.94)	0.27 (0.17–0.36)**	104.50 ± 46.76	57.93 ± 21.12	57.90 ± 12.44
Boys (*n* = 53)					
Energy (Kcal)	1280.16 (866.98–1693.33)	0.21 (0.06–0.35)**	2676.07 ± 828.09	1831.75 ± 467.13	1831.43 ± 170.58
Protein (g)	42.25 (26.38–60.13)	0.23 (0.04–0.42)**	86.33 ± 27.52	63.13 ± 19.31	63.11 ± 6.33
Carbohydrate (g)	180.85 (127.01–234.68)	0.17 (0.03–0.31)*	370.61 ± 124.47	243.25 ± 64.53	247.61 ± 20.66
Fat (g)	40.32 (24.15–56.49)	0.29 (0.14–0.45)**	98.73 ± 33.89	69.39 ± 21.19	69.42 ± 8.13
Saturated fat (g)	16.37 (11.69–21.05)	0.18 (0.05–0.32)**	32.48 ± 13.84	22.33 ± 7.00	22.1 ± 2.53
MUFA (g)	11.13 (4.84–17.42)	0.38 (0.21–0.55)**	35.02 ± 11.67	24.46 ± 13.84	24.44 ± 4.43
PUFA (g)	11.17 (6.40–15.94)	0.20 (−0.01–0.41)	20.54 ± 9.95	15.30 ± 7.67	15.30 ± 2.00
*Trans*-fatty acid (g)	0.07 (0.03–0.11)	0.12 (−0.01–0.38)	0.16 ± 0.14	0.10 ± 0.11	0.10 ± 0.03
Calcium (mg)	542.27 (352.06–732.47)	0.14 (−0.02–0.31)	1079.04 ± 429.39	695.36 ± 257.79	695.49 ± 60.97
Iron (mg)	5.45 (2.60–8.29)	0.43 (0.29–0.56)**	19.54 ± 7.48	13.78 ± 4.83	13.77 ± 3.19
Fiber (g)	11.22 (7.67–14.78)	0.19 (0.05–0.33)**	23.68 ± 9.44	15.76 ± 5.03	15.77 ± 1.81
Sugar (g)	58.13 (42.08–74.17)	0.09 (−0.03–0.21)	123.30 ± 47.70	69.04 ± 21.05	84.02 ± 10.02

*p-value < 0.05; **p-value < 0.01

In order to evaluate the reproducibility of the developed FFQ, energy and nutrients intakes obtained from FFQ-1 were compared to those from FFQ-2 using ICC, Spearman’s correlation, κw and percent agreement in quartile classification of dietary intake (same or adjacent quartiles) (Table 4). The ICC coefficients were greater than 0.5 for energy and most of the nutrients considered (*p* < 0.01) (except for *trans*-fatty acids where ICC was 0.31). The energy adjusted Spearman’s r between FFQ-1 and FFQ-2 were statistically significant at *p* < 0.001 for energy and all nutrients with the highest correlation observed for energy (0.77) and the lowest for *trans*-fatty acid (0.49). κw ranged between 0.41 for *trans*-fatty acids to 0.69 for fat, indicating a moderate to substantial level of agreement. Regarding agreement in quartile classification of FFQ-1 and FFQ-2, over 80 % of study participants were classified in the same or adjacent quartile for energy and the selected nutrients.

**Table 4:** Mean ± (SD), mean difference, Intraclass Correlation Coefficients (ICC), Spearman correlation coefficients, weighted kappa (κw) and percent agreement for energy and nutrients as measured using FFQ-1 and FFQ-2 (*n* = 111)

Nutrients	Mean FFQ-1	Mean FFQ-2	ICC	Spearman’s *r* ^a^	κw	Percent agreement (same & adjacent quartile)
Energy (Kcal)	2560.86 ± 822.32	2421.20 ± 793.40	0.71**	0.77**	0.61	92.79
Protein (g)	81.71 ± 25.88	81.03 ± 30.33	0.65**	0.65**	0.51	84.68
Carbohydrate (g)	351.87 ± 120.07	330.39 ± 113.17	0.68**	0.64**	0.62	93.69
Fat (g)	96.47 ± 35.70	89.81 ± 33.39	0.71**	0.65**	0.69	91.89
Saturated fat (g)	30.17 ± 12.47	28.25 ± 10.53	0.70**	0.57**	0.52	90.99
MUFA (g)	34.87 ± 13.31	32.54 ± 13.26	0.69**	0.56**	0.59	92.79
PUFA (g)	20.80 ± 10.35	18.96 ± 8.85	0.64**	0.55**	0.55	90.09
*Trans*-fatty acid (g)	0.16 ± 0.16	0.14 ± 0.13	0.31**	0.49**	0.41	85.58
Calcium (mg)	996.76 ± 365.68	975.27 ± 364.05	0.73**	0.67**	0.51	90.09
Iron (mg)	19.09 ± 7.15	18.05 ± 7.82	0.70**	0.68**	0.55	90.09
Fiber (g)	23.36 ± 9.60	20.51 ± 8.26	0.58**	0.65**	0.55	93.69
Sugar (g)	113.47 ± 47.94	99.17 ± 39.70	0.62**	0.52**	0.58	92.79

**P value < 0.01

^a^Adjustment for energy was carried using the residual method

## Discussion

Compared with other dietary assessment methods, FFQs are the most practical and cost-effective means for assessing diet in large-scale nutritional epidemiology studies [32]. However, due to the fact that food availability, accessibility and preferences can vary greatly between settings, it has been recommended that questionnaires be developed and validated specifically for the populations they are intended to be used for in order to produce valid and reliable data [9]. In this study, the validity and reproducibility of an Arabic FFQ to be used amongst school-aged children were examined, with the reference method being four repeated 24-HRs. Ideally, the use of a biochemical indicator or marker, such as doubly labeled water, should be adopted in validation studies as it gives an independent measure of validity for dietary intake. However, biochemical measures are expensive, require the use of sophisticated laboratories and equipment, and/or do not provide information on all the nutrients of interest [33]. Hence, researchers tend to rely on robust dietary assessment methods in the validation of nutritional tools. The dietary record approach, typically considered as the gold standard [10, 33], is a burdensome method requiring a high level of motivation for the participant to write down all the foods and their corresponding portions throughout the study period [33]. In the present study, the 24-HR was chosen as the reference method given that this approach is not limited by literacy or motivation level [9, 33]. Through bypassing these two drawbacks, the 24-HR was found to be particularly helpful in investigating dietary intakes amongst youth [33]. The 24-HR has been validated against more lengthy dietary assessment methods and in most cases found to represent participants’ dietary intakes [33–35]. Mullenbach et al. [36] found that, compared to a 3-day food record, 24-HRs administered by phone to a group of adolescents reasonably assessed mean nutrients’ intakes. Moreover, a systematic review conducted by Burrows et al. [37] reported that when compared to doubly labeled water, the 24-HR MPR approach is the most accurate dietary assessment method to estimate total energy intake in children, when conducted for at least 3 days, including weekdays and weekends and using proxies for reporting. In the present study, we used 4 days of 24-HRs, which exceeds the generally accepted minimum number of three recalls needed to represent usual intake [38].

The results of this study showed that the estimated correlations of energy-adjusted data between the FFQ and the 24-HRs were low to moderate, ranging between 0.26 for MUFA to 0.45 for iron. These results are in agreement with those reported by previous studies [29, 39–42]. In a review of 227 studies examining the validity of FFQs, mean correlation estimates for macronutrient intakes as assessed by the FFQs and the respective reference methods ranged between 0.39 for vitamin A and 0.55 for calcium, a range similar in magnitude to what was obtained in the present study [14]. However higher correlation estimates were reported by other FFQ validation studies, reaching as high as 0.85 [43–45]. The observed discrepancies in validation results between the various studies could be due to differences in study protocols (type of the FFQ, sample size, specific nutrients examined, use of reference method, recall period or number of recorded days); study populations as well as between-person variations [46].

The observed moderate agreement between the FFQ and the 24-HRs in this study could be due to the fact that dietary intake, among children, is characterized by high day-to-day variability [47, 48]. Furthermore, errors in portion size estimation and limitations of recall ability could also have contributed to the moderate agreement between the two methods. Increasing the number of recall days has been suggested to enhance this agreement. However, long recall periods may reduce the accuracy of assessment, owing to increasing fatigue and boredom, potential alterations of dietary habits and increasing likelihood of drop-outs [49].

Our results showed an overestimation of dietary intake by the FFQ as compared to the 24-HRs (mean difference was positive for energy and all the nutrients examined). This tendency of the FFQ to overestimate dietary intake was also reported by previous studies in both the adult and pediatric populations [7, 38, 41, 45, 47–49]. Such an overestimation could be due to the large number of foods listed under each food group in the FFQ, thus providing wider selection options, as compared to 24-HRs [50]. Similarly, potential inaccurate subject reporting of frequency of consumption and/or the amount of commonly consumed foods could be an additional source for this overestimation [51].

The overestimation of energy and nutrient intakes by the FFQ as compared to the 24-HRs and the moderate agreement between the two methods were further confirmed by the results of the Bland-Altman analysis, which showed a positive mean difference for energy and macronutrients. Interestingly, the Bland-Altman plots showed that mean differences between the two methods were greater at higher levels of intake. These findings are in line with those reported by previous studies [43, 52] whereby over-reporting of energy intakes were described at higher intake levels, while under-reporting was observed at lower intakes. These results suggest that the FFQ is able to estimate total energy intake better on a group level as compared to the individual level.

The derivation of the calibration coefficients in this study lead to the correction of the mean nutrient intake values that were estimated by the FFQ. In fact, the calibrated values were close to the estimates obtained by the 24 HRs. This finding is in agreement with Araujo and colleagues [29] who also found that the calibrated values were similar to the means estimated by the reference method. The range of calibration factors obtained in this study is similar in magnitude to those reported by other studies. For instance, calibration factors estimated by Araujo and colleagues [29] were found to range between 0.15 for energy and 0.48 for protein intakes; while those estimated by Voci and colleagues were found to range between 0.07 for iron and 0.40 for vitamin C [53]. Slater et al. reported higher calibration factors, varying between 0.89 for energy, 0.41 for carbohydrates, 0.22 for total fat and 0.20 for protein [54]. Calibration factors with values closer to one indicate that dietary estimates obtained by the FFQ are closer to those estimated by the reference method [55]. It is important to note that the calibrated values displayed a considerable reduction in the data dispersion as compared to the original estimates, a phenomenon also observed in other studies and which could be related to the linear relationship between the FFQ and the dietary recalls [53, 54]. Nevertheless, calibration factors are useful in correcting biases in food intake estimates, particularly when dietary intake is the exposure variable in a study of the association between diet and disease [56, 57].

In order to evaluate reproducibility, the FFQ was administered twice, four weeks apart, minimizing potential temporal changes in children’s dietary intake. The Intraclass correlation coefficients between the two FFQ administrations were between 0.31 for *trans*-fatty acids to 0.73 for calcium, a range similar to the results other reproducibility studies of FFQs in this age group [7, 58, 59]. According to Cade et al. [12] and Willet [10], such a range is considered adequate.

A major strength in the present study is the extensive process adopted to tailor the FFQ items specifically to Lebanese children and their food culture. Furthermore, the portion size section in the FFQ was designed to minimize burden on the participants and provided visual assistance with real size photos. The FFQ was interviewer-administered to ensure adequate completion [9], provide immediate feedback and checking, and minimize possible bias due to lack of understanding of the process or misinterpretation of portion sizes and intake frequencies [14]. However, the results of this study ought to be considered in light of a few limitations. First, the use of proxy in assessment of dietary intake may have introduced a source of uncertainty or error. For example, the mother may not be fully aware of all the food items eaten by her child, especially foods consumed outside the house (e.g., in school) [49]. However, during all data collection (FFQs and 24-HR), the research dietitians made sure that the child was present with his/her mother, which gave the opportunity for the mother to consult with the child regarding foods not consumed at home. Furthermore, the social desirability bias could not be ruled out as parents usually tend to overestimate intakes of food considered ‘healthy’ and underestimate less ‘healthy’ foods [14, 60]. In order to minimize such a bias, interviewers were trained to limit any judgmental verbal and non-verbal communication during the completion of the 24-HRs and FFQs. Second, many FFQ validation studies conducted among children and adolescents have used the food record method as the gold standard [45, 61, 62]. However, among populations with low schooling levels and consequently a high illiteracy rate, the use of the food record method becomes challenging and may lead to a selection bias [63]. In this study 26 % of the participating mothers had less than high school level of which 23.4 % had intermediate school level or less than. Therefore, in the context of this study, the 24-HR was the method of choice to be used as reference. The MPR approach used in this study to collect the 24-HRs minimized memory bias and standardized the interviews by using five probing stages [64]. Another limitation that ought to be considered is the use of the USDA database, as opposed to a locally developed database. However, in the context of the study, there exists no Lebanese food composition database. For traditional Lebanese dishes, a specific food composition table developed for Middle Eastern foods was used [26]. Though the use of USDA database could have led to miscalculation and errors of nutrients intakes estimations, it is less likely to have affected the reliability and validity measures of the developed FFQ since this database was used in the analysis of nutrient intakes of both the FFQ as well as the 24-HRs. Related to the dietary assessment method, is the limitation of seasonal variation, which poses the possibility of a potential error due to seasonal inconsistency of food intake [65]. In this study, in order to minimize the errors of seasonal variation, mothers were encouraged to indicate the consumption of fruits and seasonal foods and as such adjustments in calculation of nutrients intake was carried out. In addition, data collection took place in various seasons of the year to capture a wider spectrum of dietary intake across seasons. It remains important to note that a self-selection bias could have led to the over representation of the overweight and obese children in the study sample. In fact, the latest national estimate of overweight and obesity prevalence among children 6–9 years old was 36 % [22] versus 52 % obtained in this study. Such a self-selection bias could be explained by the fact that mothers of heavier children may be keener to participate in nutrition-related studies in the hope to learn more about nutrition and good dietary practices.

## Conclusion

To our knowledge, this is the first study to provide evidence for the validity and reproducibility of an FFQ to be used for the evaluation of dietary intake in a sample of Arabic speaking children. The results of this study showed that the developed FFQ, when used with calibration factors, is a useful tool in estimating dietary intake during the past year in this population. These findings fill a critical knowledge gap in the assessment of nutritional status in this young age group, and hence are important in light of the documented associations between food intake and obesity as well as the influence of childhood dietary intake on chronic disease risk in adulthood [66, 67]. The developed FFQ may be used in population-based studies aiming at monitoring dietary intakes and food consumption patterns amongst young children and at guiding the development of effective, evidence-based public health strategies for optimal growth and heath amongst children. Further research should examine the developed FFQ’s effectiveness among other paediatric groups in neighbouring countries of the region.

## Additional file

Additional file 1:
**Tables describing additional analyses that include an extensive list of nutrients.** (DOCX 44 kb)
