# Supplementary material for: Validity and reliability of a food frequency questionnaire to estimate dietary intake among Lebanese children
Source: Nutr J. 2016 Jan 12;15:4. doi: 10.1186/s12937-015-0121-1 (PMC4709981; doi:10.1186/s12937-015-0121-1)
Supplement: Additional file 1: — Tables describing additional analyses that include an extensive list of nutrients. (DOCX 44 kb) [file 12937_2015_121_MOESM1_ESM.docx]

**Additional file 1: Tables describing additional analyses that include an extensive list of nutrients.**

**Table 2.** Mean ± SD, mean difference, Spearman’s correlation (*r*) and 95% Limits of Agreement (LOA) for selected energy and nutrients intakes as measured by FFQ-1 and mean 24-HRs (n=111).

| **Nutrients** | **FFQ-1**  Mean ± SD | **24-HRs**  Mean ± SD | **Mean difference** | **Spearman’s *r***^a^ | **95% LOA** | |
| --- | --- | --- | --- | --- | --- | --- |
| Cholesterol (mg) | 198.09 ± 89.21 | 136.79 ± 69.47 | 61.30 ± 101.85 | 0.13 | -61.91 | 46.44 |
| Sodium (mg) | 3106.38 ± 1263.80 | 2024.23 ± 758.64 | 1082.15 ± 1186.70 | 0.17 | -1316.98 | -87.89 |
| Potassium (mg) | 3168.98 ± 1178.32 | 2112.75 ± 766.96 | 1056.23 ± 986.20 | 0.23* | -893.34 | 169.96 |
| Vitamin A (RE) | 1184.21 ± 2062.18 | 527.82 ± 419.26 | 656.40 ± 2096.63 | 0.28** | -1107.72 | -708.75 |
| Vitamin C (mg) | 131.09 ± 111.14 | 80.95 ± 39.79 | 50.13 ± 110.81 | 0.15 | -122.31 | -70.31 |
| Vitamin D (µg) | 5.93 ± 15.80 | 2.15 ± 2.12 | 3.79 ± 15.97 | 0.40** | -4.93 | -3.14 |
| Vitamin E (mg) | 10.10 ± 42.67 | 2.78 ± 6.76 | 7.32 ± 41.28 | 0.41** | -6.37 | -1.53 |
| Folate (µg) | 616.59 ± 689.37 | 350.72 ± 157.41 | 265.87 ± 698.19 | 0.28** | -680.67 | -487.84 |
| Vitamin K (µg) | 184. 36 ± 148.27 | 139.00 ± 206.71 | 45.36 ± 241.38 | 0.08 | 71.58 | 207.68 |
| Iodine (µg) | 25.29 ± 126.94 | 2.35 ± 12.00 | 22.93 ± 127.11 | 0.36** | -8.67 | 0.54 |
| Vitamin B 12 (µg) | 6.85 ± 15.81 | 3.20 ± 3.32 | 3.65 ± 16.17 | 0.24** | -7.02 | -4.12 |
| Phosphorus (mg) | 1299.81 ± 413.37 | 895.40 ± 268.68 | 404.41 ± 384.65 | 0.38** | -492.04 | 19.62 |
| Magnesium (mg) | 339.22 ± 113.37 | 218.95 ± 66.41 | 120.26 ± 101.86 | 0.31** | -135.85 | -13.91 |
| Zinc (mg) | 11.83 ± 8.96 | 7.74 ± 2.52 | 4.09 ± 8.90 | 0.12 | -13.26 | -9.34 |
| Copper (mg) | 1.59 ± 0.60 | 1.02 ± 0.32 | 0.57 ± 0.54 | 0.22* | -0.76 | -0.20 |
| Manganese (mg) | 3.27 ± 1.26 | 2.23 ± 0.83 | 1.05 ± 1.20 | 0.39** | -1.23 | 0.11 |
| Fluoride (µg) | 544.32 ± 1114.52 | 296.58 ± 370.54 | 247.74 ± 1073.33 | 0.37** | -472.69 | -188.19 |

** *P* value < 0.01
^a^ Adjustment for energy was carried using the residual method ; RE: Retinol Equivalent

**Table 3**. Calibration parameters and means ± (SD) for energy and nutrients intake estimated from FFQ-1, 24-HRs and calibrated FFQ-1 for boys and girls (n=111).

| **Energy/nutrients** | **α (95% CI)** | **Β** **(95% CI)** | **FFQ-1**  Mean ± SD | **24-HRs**  Mean ± SD | **Calibrated FFQ-1**  Mean ± SD |
| --- | --- | --- | --- | --- | --- |
| **Females (n=58)** | | | | | |
| Cholesterol (mg) | 96.36 (47.77-144.94) | 0.21 (-0.03-0.26) | 182.01±85.01 | 135.51±19.07 | 135.49±18.28 |
| Sodium (mg) | 1015.07 (481.06-1549.08) | 0.30 (0.13-0.47)** | 2911.76±1061.68 | 1894.03±755.39 | 1894.42±320.63 |
| Potassium (mg) | 644.18 (264.76-1023.60) | 0.437 (0.32-0.55)** | 3124.96±1241.34 | 2010.61±754.36 | 2009.79±542.46 |
| Vitamin A (RE) | 504.46 (359.59-649.32) | 0.001 (-0.05-0.05) | 1374.47±2727.90 | 506.31±486.65 | 503.08±2.73 |
| Vitamin C (mg) | 73.11 (59.72-86.49) | 0.04 (-0.23-0.11) | 136.63±139.55 | 78.49±36.29 | 78.57±5.58 |
| Vitamin D (µg) | 1.84 (1.23-2.45) | -0.005 (-0.03-0.02) | 6.41±20.86 | 1.81±2.20 | 1.81±0.10 |
| Vitamin E (mg) | 6.21 (4.69-7.74) | 0.03 (-0.003-0.07) | 12.75±56.24 | 2.94±8.09 | 6.75±1.29 |
| Folate (µg) | 327.64 (277.79-377.50) | 0.003 (-0.04-0.05) | 525.88±626.47 | 320.56±440.90 | 329.56±2.71 |
| Vitamin K (µg) | 3.42 (2.21-4.63) | -0.005 (-0.06-0.05) | 7.54±20.88 | 3.38±4.30 | 3.38±0.10 |
| Iodine (µg) | 414.05 (216.62-611.47) | 0.35 (0.20-0.50)** | 1235.33±379.03 | 847.31±254.08 | 847.64±133.06 |
| Vitamin B 12 (µg) | 89.80 (45.20-134.41) | 0.09 (-0.8-0.25) | 203.05±181.04 | 107.29±112.39 | 107.27±15.57 |
| Phosphorus (mg) | 2.69 (-1.22-6.61) | 0.01 (-0.02-0.02) | 33.09±167.23 | 2.71±14.46 | 2.69±0.01 |
| Magnesium (mg) | 116.68 (76.95-156.40) | 0.27 (0.16-0.38)** | 334.49±122.43 | 206.67±60.71 | 206.73±32.93 |
| Zinc (mg) | 6.88 (5.97-7.80) | 0.05 (-0.01-0.10) | 11.85±11.56 | 7.43±2.45 | 7.43±0.53 |
| Copper (mg) | 0.61 (0.40-0.81) | 0.24 (0.12-0.36)** | 1.59±0.67 | 0.99±0.34 | 0.99±0.16 |
| Manganese (mg) | 1.35 (0.84-1.85) | 0.23 (0.09-0.38)** | 3.26±1.36 | 2.11±0.79 | 2.11±0.32 |
| Fluoride (µg) | 93.39 (-27.65-214.47) | 0.43 (0.28-0.58)** | 525.88±626.47 | 320.56±440.90 | 320.56±270.63 |
| **Males (n=53)** | | | | | |
| Cholesterol (mg) | 117.49 (76.21-158.76) | 0.10 (-0.08-0.27) | 215.70±91.15 | 138.19±57.90 | 138.19±170.58 |
| Sodium (mg) | 1558.59 (1066.29-2050.90) | 0.18 (0.05-0.32)** | 3319.35±1433.41 | 2166.71±743.24 | 2166.03±262.31 |
| Potassium (mg) | 1414.91 (798.07-2031.75) | 0.252 (0.70-0.43)* | 3217.15±1115.11 | 2224.52±772.14 | 2225.63±281.01 |
| Vitamin A (RE) | 508.74 (369.28-648.23) | 0.04 (-0.06-0.15) | 976.01±868.50 | 551.35±333.40 | 551.68±38.21 |
| Vitamin C (mg) | 58.60 (34.58-82.66) | 0.2 (0.03-0.37) | 125.02±68.62 | 83.65±43.49 | 83.60±13.72 |
| Vitamin D (µg) | 2.32 (1.63-3.02) | 0.03 (-0.04-0.011) | 5.41±7.11 | 2.51±1.99 | 2.51±0.25 |
| Vitamin E (mg) | 5.00 (3.67-6.31) | 0.13 (0.05-0.20)** | 7.20±19.18 | 2.60±4.99 | 6.54±1.69 |
| Folate (µg) | 309.87 (219.84-399.89) | 0.108 (-0.02-0.24) | 592.57±330.36 | 373.99±160.32 | 373.86±35.68 |
| Vitamin K (µg) | 2.82 (2.18-3.45) | 0.03 (-0.04-0.10) | 6.20±7.08 | 3.00±1.72 | 3.00±0.21 |
| Iodine (µg) | 679.22 (438.13-920.327) | 0.20 (0.03-0.37)* | 1370.38±440.71 | 948.03±276.65 | 947.82±86.38 |
| Vitamin B 12 (µg) | 87.90 (-57.37-233.17) | 0.52 (-0.24-1.28) | 163.91±98.79 | 173.72±272.38 | 173.79±51.77 |
| Phosphorus (mg) | 1.53 (-0.94-4.01) | 0.03 (-0.01-0.07) | 16.75±57.37 | 1.98±8.67 | 1.97±1.49 |
| Magnesium (mg) | 144.40 (80.95-207.86) | 0.26 (0.08-0.43)* | 344.06±103.50 | 232.40±70.25 | 232.48±26.49 |
| Zinc (mg) | 7.51 (5.62-9.40) | 0.05 (-0.10-0.20) | 11.82±4.85 | 8.08±2.57 | 8.09±0.24 |
| Copper (mg) | 0.66 (0.42-0.91) | 0.24 (0.09-0.40) | 1.59±0.52 | 1.04±0.30 | 1.05±0.13 |
| Manganese (mg) | 1.36 (0.71-2.12) | 0.30 (0.12-0.49)** | 3.30±1.16 | 2.36±0.85 | 2.36±0.35 |
| Fluoride (µg) | 256.01 (174.53-337.49) | 0.025 (-0.26-0.07) | 564.49±1482.11 | 270.33±275.71 | 270.12±275.71 |

^*^ *p-*value < 0.05; ^**^ *p-*value < 0.01

**Table 4**. Mean ± (SD), mean difference, Intraclass Correlation Coefficients (ICC), weighted kappa (κw) and percent agreement for energy and nutrients as measured using FFQ-1 and FFQ-2 (n=111).

| **Nutrients** | **Mean FFQ-1** | | **Mean FFQ-2** | **ICC** | **κw** | **Percent agreement**  *(same & adjacent quartile)* |
| --- | --- | --- | --- | --- | --- | --- |
| Cholesterol (mg) | | 198.09 ± 89.21 | 193.35 ± 102.11 | 0.708** | 0.49 | 90.09 |
| Sodium (mg) | | 3106.38 ± 1263.80 | 3113.54 ± 1322.27 | 0.750** | 0.58 | 89.19 |
| Potassium (mg) | | 3168.98 ± 1178.32 | 2906.94 ± 1170/64 | 0.647** | 0.55 | 92.79 |
| Vitamin A (RE) | | 1184.21 ± 2062.18 | 971.18 ± 894.40 | 0.117 | 0.43 | 71.17 |
| Vitamin C (mg) | | 131.09 ± 111.14 | 113.61 ± 68.93 | 0.290** | 0.54 | 90.99 |
| Vitamin D (µg) | | 5.93 ± 15.80 | 4.85 ± 6.70 | 0.086 | 0.56 | 83.78 |
| Vitamin E (mg) | | 10.10 ± 42.67 | 7.47 ± 17.66 | 0.107 | 0.48 | 86.49 |
| Folate (µg) | | 616.59 ± 689.37 | 552.31 ± 350.94 | 0.206* | 0.58 | 90.09 |
| Vitamin K (µg) | | 6.85 ± 15.81 | 5.90 ± 6.83 | 0.098 | 0.46 | 88.29 |
| Iodine (µg) | | 1299.81 ± 413.37 | 1268.19 ± 456.46 | 0.630** | 0.55 | 92.79 |
| Vitamin B 12 (µg) | | 184. 36 ± 148.27 | 155.07 ± 97.92 | 0.573** | 0.58 | 95.50 |
| Phosphorus (mg) | | 25.29 ± 126.94 | 18.04 ± 50.80 | 0.086 | 0.67 | 87.39 |
| Magnesium (mg) | | 339.22 ± 113.37 | 315.61 ± 114.00 | 0.558** | 0.58 | 90.09 |
| Zinc (mg) | | 11.83 ± 8.96 | 11.30 ± 5.70 | 0.291** | 0.54 | 90.99 |
| Copper (mg) | | 1.59 ± 0.60 | 1.42 ± 0.50 | 0.578** | 0.57 | 90.09 |
| Manganese (mg) | | 3.27 ± 1.26 | 3.04 ± 1.18 | 0.661** | 0.61 | 91.89 |
| Fluoride (µg) | | 544.32 ± 1114.52 | 414.26 ± 638.12 | 0.520** | 0.60 | 93.69 |

** *P* value < 0.01

RE: Retinol Equivalent
